# AN 11-YEAR-OLD BOY WITH DARK SKIN, SWALLOWING DIFFICULTY AND ABSENCE OF TEARS

**DOI:** 10.4103/0019-5154.49007

**Published:** 2009

**Authors:** Debkrishna Mallick, Rajoo Thapa

**Affiliations:** *From the Department of Pediatrics, The Institute of Child Health, Kolkata, West Bengal, India*

An 11-year-old boy born to nonconsanguineous parents was initially seen for complaint of progressive darkening of his body along with blackish pigmentation of tongue for the last 4 years, and easy fatigability with increasing weakness of the limbs for 2 years. He also complained of progressive difficulty in swallowing food, especially liquids, for the last 2 years. There was history of a male sibling death at the age of 9 months due to unknown causes. The past medical history of the boy was noncontributory.

On examination his weight and height were 21 kg and 124 cm respectively (both below age and sex adjusted 3^rd^ percentile; Agarwal KN *et al.*, Indian Academy of Pediatrics, 2007). The vitals were normal and mild pallor was noted. Diffuse hyperpigmentation was noted over the face [Figure 1] and flexural areas, including the axillae, anterior portion of the neck, groins, genitals and the back of knee. Darkening and desiccation of the skin was also significant over the knuckles of the fingers of both the hands [Figure 2]. The tongue was remarkable for blackish brown, leathery hyperpigmentation, especially prominent over the posterior two thirds and the lateral borders [Figure 3]. The tip was uninvolved. The penis was normal in size; with bilaterally distended testes, each 2.5 mL in volume. Neurological examination revealed weakness in the proximal muscles of both upper and lower limbs. The findings from local examination of the neck, including the thyroid, were normal. There was no evidence of organomegaly. The findings from examination of the other systems were normal. On direct questioning, the mother stated that her son had no tears even when he cried.

**Figure 1:**
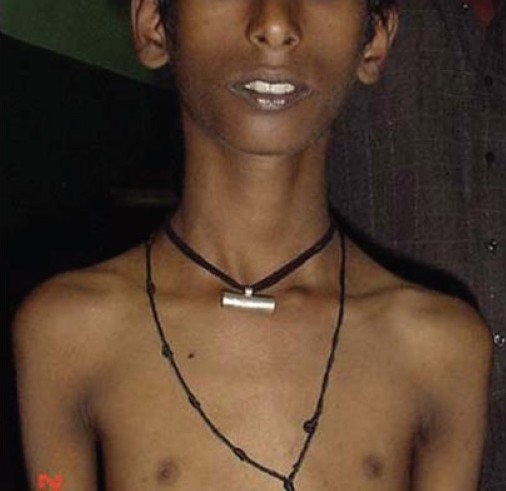
Diffuse facial and axillary hyperpigmentation

**Figure 2:**
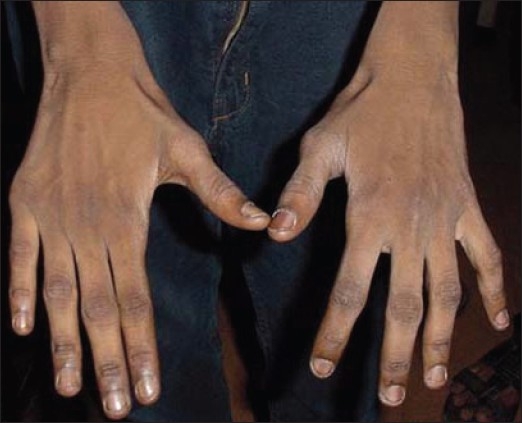
Knuckle hyperpigmentation

**Figure 3:**
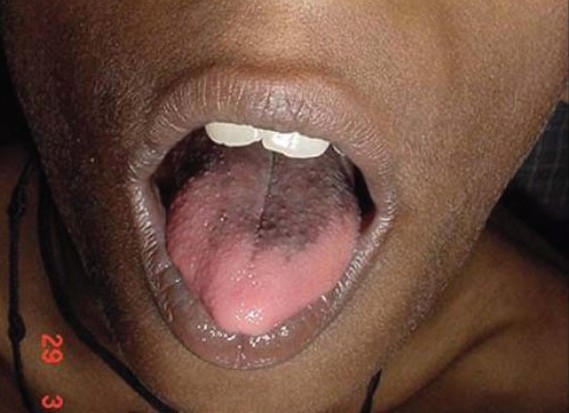
Tongue hyperpigmentation

The blood glucose, renal function tests, liver function tests and the complete blood count were normal; besides blood hemoglobin, which was 9.8 g/dL. Serum sodium and potassium were 141 mEq/L and 5.4 mEq/L respectively. The basal serum cortisol level from a blood sample taken at 8 am was < 10 mg/dL (normal >20 mg/dL). Prolonged adrenocorticotropic hormone (ACTH) stimulation test (1 mg Synacthen depot IM daily for 3 days) confirmed the decreased adrenal reserves. A barium esophagogram was performed, which is depicted in Figure 4. Schirmer's test, using Whatman no. 41 filter paper, for assessment of total (basic + reflex) tear secretion showed a value of < 5 mm.

**Figure 4:**
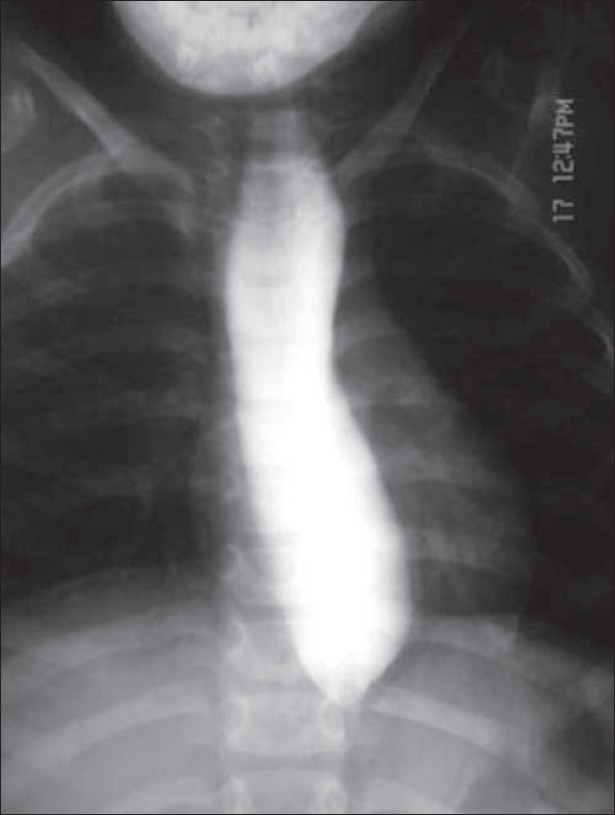
Barium esophagogram of the patient

## Questions

1. What is the diagnosis?
2. What are the different physical manifestations of this condition?
3. What are the differential diagnoses?

## Answers

1. Allgrove's, or “3A”, syndrome. It is an autosomal recessive disorder characterized by alacrima, achalasia and ACTH insensitivity. In 1978, Allgrove *et al.*[1] described two unrelated pairs of siblings with isolated glucocorticoid deficiency and achalasia of the esophagus. The latter condition involved delayed passage of food into the stomach and consequent dilation of the thoracic esophagus. Three of these individuals also had defective tear production, leading the authors to speculate that the combination of achalasia, adrenal deficiency and alacrima represented an inherited familial disorder. Following this, a number of authors published similar reports that have helped to define the primary and associated features of this syndrome. Several authors published descriptions of a more global autonomic disturbance associated with the original Allgrove triad, leading one author to suggest the name “4A” syndrome (adrenal insufficiency, achalasia of the cardia, alacrima, autonomic abnormalities).[2,3] Recent studies have identified mutations of gene on chromosome 12q13 (WD- repeat protein termed ALADIN) responsible for this condition.[4]
2. The distinctive facial appearance of Allgrove's syndrome consists of a long, thin face with a long philtrum, narrow upper lip and a down-turned mouth. Microcephaly is a frequent association.[5] Conjunctival injection (means redness) and irritation may be the only obvious signs of alacrima. Slit lamp examination may reveal punctate keratopathy or corneal ulceration. Definitive diagnosis of alacrima can be made at bedside with the Schirmer's test.[6] Cardiac examination findings may be abnormal due to a number of autonomic nervous system defects that may accompany Allgrove's syndrome. Orthostatic hypotension and diminished heart rate variations during deep breathing and Valsalva maneuver are well documented. Respiratory symptoms secondary to recurrent aspiration pneumonitis as a consequence of esophageal dysfunction may be seen. Neurological manifestations include hyperreflexia, dysarthria, hypernasal speech with palatopharyngeal incompetence, and ataxia. Adults may exhibit progressive neural degeneration, develop parkinsonian features and show mental deterioration.[7] Patients with severe degrees of ACTH insensitivity may present with features of circulatory shock.
  Skin examination of patients may reveal abnormal findings that assist in confirming diagnosis. Hyperpigmentation is common but may be observed less frequently than in other forms of primary adrenal failure. Hyperkeratosis and fine fissuring of the palms of the hands and soles of the feet represent a unique set of features of this syndrome.[6]
3. Differentials of Allgrove's syndrome include the following:
  1. Familial glucocorticoid deficiency (FGD), which has adrenal insufficiency as the only manifestation.[8] The gene for ACTH receptor, which is responsible for FGD, has been found on 18p11.2.
  2. Adrenoleukodystrophy (ALD), an X-linked recessive white matter disorder, causes impaired glucocorticoid production with minimal or no mineralocorticoid production.[9]
